# 

**Published:** 1808-03

**Authors:** 


### NEW MEDICAL PUBLICATIONS.

A Treatise on Pulmonary Consumption, by James Saunders, M. D. Edinburgh. 8vo. 8s. 6d. boards.

A Pamphlet describing the fatal Effects of the Cow-Pox, manifested by a Narrative of the Occurrences which have recently happened at Ringwood, in Hampshire. 1s. 6d.

An authentic Account of the Ringwood Cases, in a Letter to Dr. Jenner: being a Defence of Vaccination, against several unfair and insidious Attacks of John Birch, Esq.; and containing a Report of the Royal Jennerian Society, after a careful and public Investigation of those Cases upon the Spot.

Debates in Parliament, respecting the Jennerian Discovery, with the Report of the College of Physicians on the Vaccine Inoculation, by Charles Murray. 5s.

